# Patient outcomes following home-based outpatient parenteral antimicrobial therapy and facility-based outpatient parenteral antimicrobial therapy: a systematic review and meta-analysis

**DOI:** 10.1017/ash.2023.458

**Published:** 2023-10-19

**Authors:** Shinya Hasegawa, Joseph Tholany, Heather Healy, Hiroyuki Suzuki

**Affiliations:** 1 Center for Access & Delivery Research & Evaluation (CADRE), Iowa City Veterans Affairs Health Care System, Iowa City, IA, USA; 2 Division of Infectious Diseases, Department of Internal Medicine, University of Iowa, Iowa City, IA, USA; 3 Hardin Library for the Health Sciences, University of Iowa Libraries, Iowa City, IA, USA

## Abstract

In this systematic literature review and meta-analysis, we did not find a statistically significant difference in readmission and treatment failure rates between home-based and facility-based OPAT. Optimal patient selection for appropriate OPAT location appears to be more important than the location itself for the best OPAT outcome.

## Introduction

Outpatient parenteral antimicrobial therapy (OPAT) has been used to treat various infections while avoiding prolonged acute-care hospital admission. However, readmission rates of OPAT patients can be higher than 20%.^1^ A few studies identified that discharge to skilled nursing homes (SNF) or long-term care facilities was associated with higher readmission rates compared to discharge to home, but these were single-center studies conducted with a small number of patients.^2,3^ It remains unclear how the OPAT location affects outcomes. This systematic literature review and meta-analysis were conducted to investigate whether there is a difference in outcomes for patients undergoing home-based OPAT (home-OPAT) compared to those undergoing facility-based OPAT (facility-OPAT).

## Methods

A systematic literature review was performed following the Preferred Reporting Items for Systematic Reviews and Meta-Analysis (PRISMA) checklist^4^ and the Meta-Analysis of Observational Studies in Epidemiology guidelines.^5^ The study protocol was registered in the International Prospective Register for Systematic Reviews (PROSPERO) database (CRD42022378497). The following databases were searched, without any date or language limits, from inception to 12 April 2023 for eligible citations: Ovid MEDLINE, Embase (Embase.com), Cochrane CENTRAL (Wiley), CINAHL (EBSCO), Web of Science Core Collection, and Scopus. Full database search strategies are available in Supplemental Table 1. A librarian (HH) designed and executed the strategies and the searches. Results were combined in EndNote and duplicates were removed through automatic and manual methods. All potentially relevant studies were screened by three reviewers (SH, JT, and HS). Original research manuscripts or abstracts which compared outcomes between home-OPAT and facility-OPAT were included. Studies that evaluated only home-OPAT or facility-OPAT were excluded. In addition, editorials, commentaries, or review articles were excluded. Authors of ten included studies were contacted to gather additional data. Using a standardized abstraction form, details of each study were compiled. If the study reported more than one type of facility-OPAT separately, we summarized all types of facility-OPATs together for the sake of simplicity. The primary outcome evaluated was readmission rates, and the secondary outcomes were treatment failure, laboratory results monitoring, and infectious diseases (ID) follow-up rates. Data abstraction for each study was conducted by two of three reviewers. The risk of bias was assessed using the Downs and Black scale. Inconsistent assessments were resolved by discussion and judged by the third reviewer.

The random-effects models with inverse variance weighting were used to estimate the pooled odds ratio (OR) and 95% confidence interval. Heterogeneity between studies was assessed with *I* ^2^ estimation and the Cochran’s Q statistic test. Publication bias was assessed using a funnel plot. The Cochrane Review Manager (RevMan) version 5.4 was used for statistical analyses.

## Results

**Figure 1.**
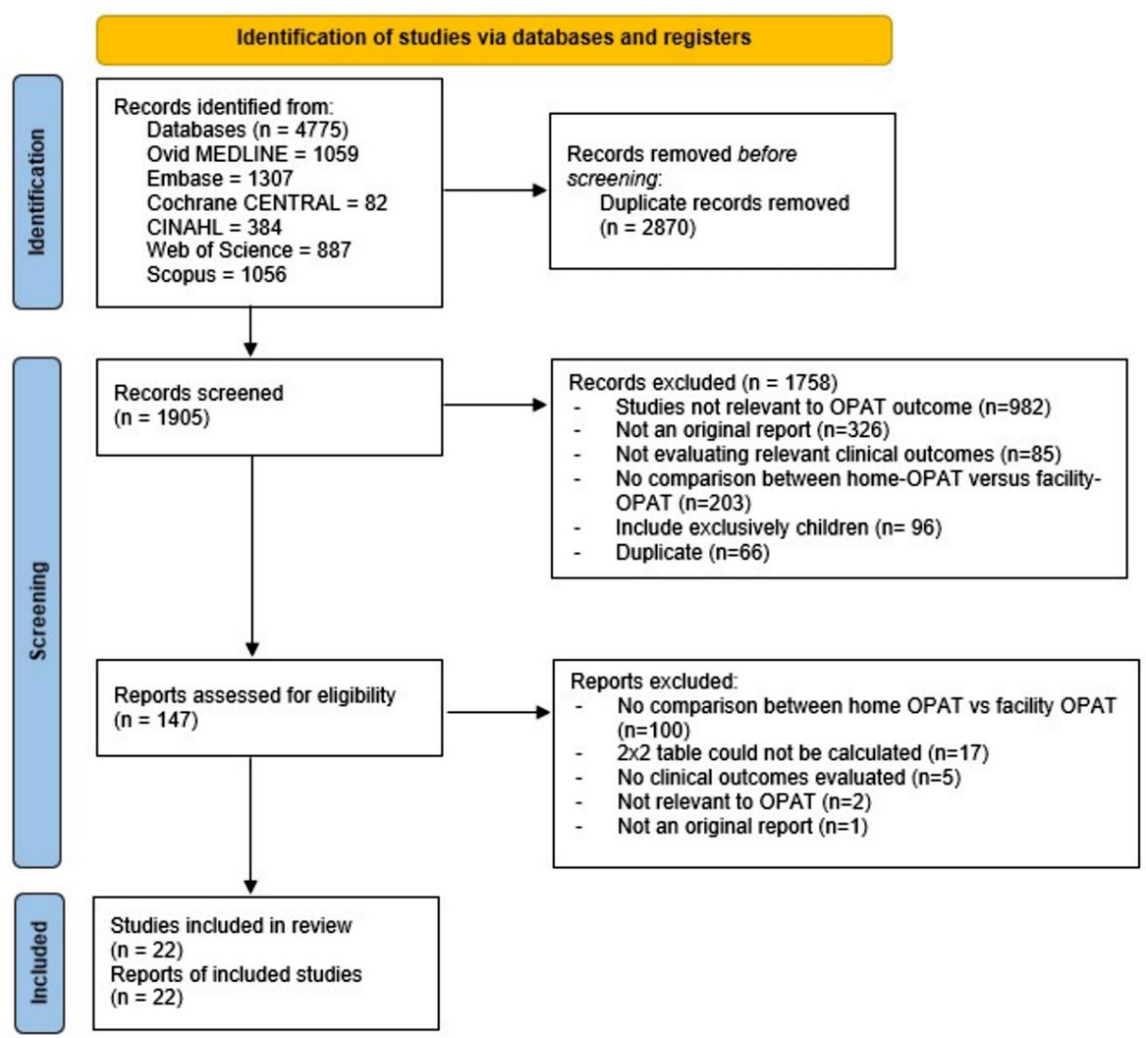
Preferred reporting items for systematic reviews and meta-analyses (PRISMA) flow diagram.

Among the 4,775 studies screened, 22 studies were included in the systematic literature review and meta-analysis (Figure 1). In total, 7,539 home-OPAT patients and 3,857 facility-OPAT patients were included. Of the 22 studies, 18 studies were retrospective cohort studies, two studies were quasi-experimental studies, and two were case-control studies (Supplemental Table 2). Twenty studies were conducted in the United States, one in the Netherlands, and one in France. Most of the studies (18/22) were single-center studies. Facility-OPAT was provided at SNF in 18 studies and at a rehabilitation center in 11 studies. A bone and joint infection was the most common indication for OPAT in 17–86%.

**Figure 2.**
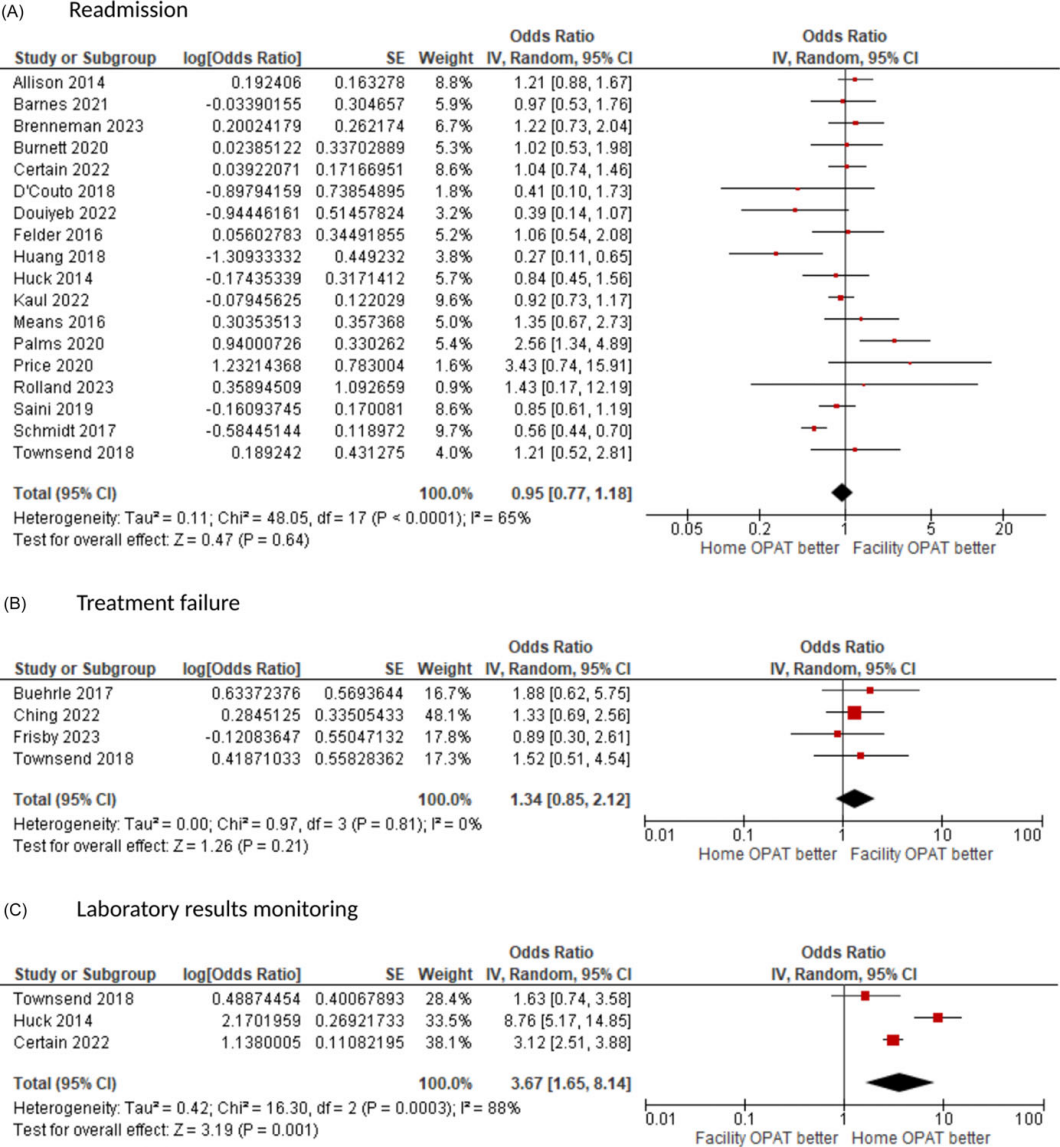
Forrest plots for readmission (A), treatment failure (B), and laboratory result monitoring (C).

Eighteen studies compared readmission rates between home-OPAT and facility-OPAT. Among them, the 30-d readmission rate was used in 11 studies, the readmission rate during the OPAT course was used in four studies, the 90-d readmission rate was used in one study, and two studies did not specify the follow-up period. When pooled together, there was not a statistically significant difference in readmission rates with an OR = 0.95 (95% CI: 0.77–1.18, *I* ^2^ = 65%) (Figure 2A). When the analysis was limited to three studies that reported multivariable logistic regression results, there was also not a statistically significant difference in readmission rates with an OR = 0.86 (95% CI: 0.26–2.82, *I* ^2^ = 88%). Among four studies that assessed treatment failures, there was not a statistically significant difference with an OR = 1.34 (95% CI: 0.85–2.12, *I* ^2^ = 0%) (Figure 2B). Lastly, among three studies that evaluated laboratory results monitoring, home-OPAT was associated with a significantly higher rate of laboratory results monitoring compared to facility-OPAT with an OR = 3.67 (95% CI: 1.65–8.14) but these studies were highly heterogenous (*I* ^2^ = 88%) (Figure 2C). Only one study reported a 21-d ID clinic follow-up rate (35.8% in home-OPAT and 31.9% in facility-OPAT, *p* = 0.11). There was little evidence of publication bias because studies were reasonably balanced around the pooled OR (Supplemental Figure).

## Discussion

In this systematic literature review and meta-analysis, we did not find a significant difference in readmission rates and treatment failure between home-OPAT and facility-OPAT. Home-OPAT may be associated with better laboratory results monitoring although the supporting studies were highly heterogenous. Our results suggest that the location of OPAT is not a single strong factor that affects OPAT outcomes.

The lack of difference in outcomes between home-OPAT and facility-OPAT can be partially explained by the variation in the quality of care provided by these facilities as well as quality of care provided by the patient/caretakers or home agencies. In addition, patients undergoing facility-OPAT may have more comorbidities or adverse complications from their infections compared to those undergoing home-OPAT.^6^ Although we did not observe a difference in outcomes when limiting our analysis to studies that performed multivariable analysis, the difference in patient-level factors between the two groups may also explain the variation in outcomes in the studies that did not perform multivariable analysis.

Our results suggest that facility-OPAT may be associated with poorer laboratory result monitoring. We did not find a link between laboratory result monitoring and outcomes although a previous study suggested that non-availability of laboratory test monitoring was associated with 2.5 times increased odds of readmission while on OPAT.^7^ This discrepancy is likely because laboratory monitoring is not a single determinant for patient outcomes but rather a process measure that could lead to improved outcomes when combined with other processes. ID clinic follow-up rate is another process measure of successful OPAT care as shown by previous studies, which suggested that it was associated with lower readmission rates.^8,9^ In our systematic literature review, one study reported similar ID clinic follow-up rates between home-OPAT and facility-OPAT although there were not enough results to conduct a meta-analysis. A bundled approach may be the best approach to improve OPAT care. At one center, a transition of care OPAT bundle involving a multidisciplinary OPAT team, patient selection, patient/family education, care transition, and outpatient care coordination decreased 30-d readmission rates from 26.1% to 13.0%.^10^ The bundled approach included evaluation of patients for an optimal location of OPAT by the multidisciplinary OPAT team. It appears that optimal patient selection for home-OPAT or facility-OPAT is more important for the improved OPAT outcomes than the OPAT location itself.

Our systematic literature review and meta-analysis have limitations. First, there was heterogeneity among studies including the location of facility-OPAT, the infections for which OPAT was indicated, the type of antimicrobials, and other patient factors. In addition, most of the studies did not adjust variables between home-OPAT and facility-OPAT. Second, we could not assess other important aspects of OPAT care such as cost or patient satisfaction due to a lack of information.

In conclusion, we did not find a significant difference in readmission and treatment failure rates between home-OPAT and facility-OPAT in our systematic literature review and meta-analysis. Optimal patient selection for appropriate OPAT location appears to be more important than the location itself for the best OPAT outcomes.

## Supporting information

Hasegawa et al. supplementary material Hasegawa et al. supplementary material

## Supplementary material

For supplementary material accompanying this paper visit http://doi.org/10.1017/ash.2023.458. click here to view supplementary material
