# Supplementary material for: Patient outcomes following home-based outpatient parenteral antimicrobial therapy and facility-based outpatient parenteral antimicrobial therapy: a systematic review and meta-analysis
Source: Antimicrob Steward Healthc Epidemiol. 2023 Oct 19;3(1):e169. doi: 10.1017/ash.2023.458 (PMC10644170; doi:10.1017/ash.2023.458)

**Supplemental Table 1.** Systematic literature search strategies

**Ovid MEDLINE**

1. exp Anti-Infective Agents/

2. exp Infusions, Parenteral/

3. outpatient*.ti,ab.

4. 1 and 2 and 3

5. (outpatient* adj4 (parenteral or intravenous or IV) adj4 (antimicrobial or anti microbial or antibiotic or anti biotic or antibacterial or anti bacterial or antiinfective or anti infective or antifungal or anti fungal or antimycotic or anti mycotic or antiviral or anti viral or antivirus or anti virus or antiparasitic or anti parasitic) adj4 (therap* or treat*)).ti,ab,kf.

6. OPAT.ti,ab,kf.

7. 4 or 5 or 6

**Embase**

#5. #1 OR #2 OR #3 OR #4

#4. opat:ti,ab,kw

#3. (outpatient* NEAR/4 (parenteral OR intravenous OR iv) NEAR/4 (antimicrobial OR 'anti microbial' OR

antibiotic OR 'anti biotic' OR antibacterial OR 'anti bacterial' OR antiinfective OR 'anti infective' OR antifungal OR 'anti fungal' OR antimycotic OR 'anti mycotic' OR antiviral OR 'anti viral' OR antivirus OR 'anti virus' OR antiparasitic OR 'anti parasitic') NEAR/4 (therap* OR treat*)):ti,ab,kw

#2. 'outpatient parenteral antibiotic therapy'/exp

#1. 'outpatient parenteral antimicrobial therapy'/exp

**Cochrane CENTRAL**

#1 MeSH descriptor: [Anti-Infective Agents] explode all trees

#2 MeSH descriptor: [Infusions, Parenteral] explode all trees

#3 (outpatient*):ti,ab,kw

#4 #1 AND #2 AND #3

#5 (outpatient* NEAR/4 (parenteral or intravenous or iv) NEAR/4 (antimicrobial or "anti microbial" or antibiotic or "anti biotic" or antibacterial or "anti bacterial" or antiinfective or “anti infective” or antifungal or "anti fungal" or antimycotic or "anti mycotic" or antiviral or "anti viral" or antivirus or “anti virus” or antiparasitic or "anti parasitic") NEAR/4 (therap* or treat*)):ti,ab,kw

#6 (OPAT):ti,ab,kw

#7 {OR #4-#6}

**CINAHL**

S1 (MH "Antiinfective Agents+")

S2 (MH "Infusions, Parenteral+")

S3 TI outpatient*

S4 AB outpatient*

S5 S3 OR S4

S6 S1 AND S2 AND S5

S7 TI outpatient* N4 (parenteral or intravenous or IV) N4 (antimicrobial or "anti microbial" or antibiotic or "anti biotic" or antibacterial or "anti bacterial" or antiinfective or "anti infective" or antifungal or "anti fungal" or antimycotic or "anti mycotic" or antiviral or "anti viral" or antivirus or "anti virus" or antiparasitic or "anti parasitic") N4 (therap* or treat*)

S8 AB outpatient* N4 (parenteral or intravenous or IV) N4 (antimicrobial or "anti microbial" or antibiotic or "anti biotic" or antibacterial or "anti bacterial" or antiinfective or "anti infective" or antifungal or "anti fungal" or antimycotic or "anti mycotic" or antiviral or "anti viral" or antivirus or "anti virus" or antiparasitic or "anti parasitic") N4 (therap* or treat*)

S9 TI OPAT

S10 AB OPAT

S11 S6 OR S7 OR S8 OR S9 OR S10

**Web of Science Core Collection**

1: AK=OPAT

2: AB=OPAT

3: TI=OPAT

4: AK=(outpatient* NEAR/4 (parenteral or intravenous or IV) NEAR/4 (antimicrobial or "anti microbial" or antibiotic or "anti biotic" or antibacterial or "anti bacterial" or antiinfective or "anti infective" or antifungal or "anti fungal" or antimycotic or "anti mycotic" or antiviral or "anti viral" or antivirus or "anti virus" or antiparasitic or "anti parasitic") NEAR/4 (therap* or treat*))

5: AB=(outpatient* NEAR/4 (parenteral or intravenous or IV) NEAR/4 (antimicrobial or "anti microbial" or antibiotic or "anti biotic" or antibacterial or "anti bacterial" or antiinfective or "anti infective" or antifungal or "anti fungal" or antimycotic or "anti mycotic" or antiviral or "anti viral" or antivirus or "anti virus" or antiparasitic or "anti parasitic") NEAR/4 (therap* or treat*))

6: TI=(outpatient* NEAR/4 (parenteral or intravenous or IV) NEAR/4 (antimicrobial or "anti microbial" or antibiotic or "anti biotic" or antibacterial or "anti bacterial" or antiinfective or "anti infective" or antifungal or "anti fungal" or antimycotic or "anti mycotic" or antiviral or "anti viral" or antivirus or "anti virus" or antiparasitic or "anti parasitic") NEAR/4 (therap* or treat*))

7: #1 OR #2 OR #3 OR #4 OR #5 OR #6

**Scopus**

( ( TITLE-ABS-KEY ( outpatient* ) ) W/4 ( TITLE-ABS-KEY ( parenteral OR intravenous OR IV ) ) W/4 ( TITLE-ABS-KEY ( antimicrobial OR "anti microbial" OR antibiotic OR "anti biotic" OR antibacterial or “anti bacterial” OR antiinfective OR "anti infective" OR antifungal OR "anti fungal" OR antimycotic OR “anti mycotic” OR antiviral OR "anti viral" OR antivirus OR "anti virus" OR antiparasitic OR “anti parasitic”) ) W/4 ( TITLE-ABS-KEY ( therap* OR treat* ) ) ) OR ( TITLE-ABS-KEY ( opat ) )

**Supplemental Table 2**. Summary of the 22 studies included in the systematic literature review.

| First author/ publication year/location | Setting | Study design/  Study population | Adjustment for confounders | Study period | Characteristic of included patients | Source of infections | Type of antibiotics used for OPAT | Outcome | D&B score |
| --- | --- | --- | --- | --- | --- | --- | --- | --- | --- |
| Allison^1^/  2014/  Massachusetts, US | Single academic center | Retrospective cohort study/  782 OPAT patients | N/A | 3 years | Adult, Mean age 58 (range 18-95)  DM 30.1%  CKD 24.2%  History of drug-resistant organism 16.6%  Immunocompromised 29.7% | BJI 20.3%  IE 10.0%  Other bacteremia 24.3%  SSTI 7.8%  UTI 13.2%  IAI 11.0%  PNA 9.5%  PJI 7.5%  CNS 4.6%  Cardiovascular device 4.3%  Epidural abscess 3.2% | Cephalosporins 25.4%  Carbapenems 19.1%  Daptomycin 5.2%  Antifungals 3.7%  Antivirals 4.7%  Anti-staphylococcal β-lactams 13.3%  FQ 8.1%  AG 5.0%  Antipseudomonal β-lactams 4.0%  Metronidazole 3.6% | 413 and 366 OPAT patients were discharged to home and facility (rehabilitation, SNF), respectively.  Unplanned 30-day readmission was 26.5% in total, 28.3% in home-OPAT group, 24.6% in facility-OPAT group. | 22 |
| Barnes^2^/  2021/  North Carolina, US | Single academic center | Case-control study/  232 OPAT patients | N/A | 1 year | *Adult, Mean age 56.4 (range 18-100)  DM 43.5%  HF 19.8%  CKD 19.8%  Decubitus ulcer 11.2%  *These numbers were for patients including 13 patients whom OPAT was administered at an infusion center | *BJI 43.5%  IE 24.5%  SSTI 5.5%  DFI 13.5%  CNS 4.4%  GI/GU 4% | N/A | 116 OPAT patients with unplanned readmission and 116 without unplanned readmission. Overall, 160 and 59 patients were discharged to home and facility (SNF), respectively. Unplanned 30-day readmission was 50.0% in total, 50.0% in home-OPAT group, 50.8% in facility-OPAT group. | 21 |
| Brenneman^3^/  2023/  North Carolina, US | Single academic center | Retrospective cohort study/  470 OPAT patients | N/A | 7 months | Adult, Mean age 50.4 (SD 16.1)  IVDU 5.1% | BJI 58.7%  Endovascular infection 13.6%  SSTI 7.0%  GU 4.9%  Respiratory 3.2% | Vancomycin 36.2%  AG 0.2% | 335 and 135 OPAT patients were discharged to home and facility (SNF), respectively.  Unplanned 30-day readmission was 20.0% in total, 20.9% in home-OPAT group, 17.8% in facility-OPAT group. | 23 |
| Buehrle^4^/  2017/  Philadelphia, US | Single academic center | Retrospective cohort study/  67 IVDUs who received OPAT | N/A | 1 year 2 months | Adult, Mean age 34.5 (range 19-63)  IVDU 100% | IE 52%  Other bacteremia 4%  SSTI 4%  Epidural abscess 7% | Penicillins 34%  Cephalosporins 16%  Vancomycin 33% | 20 and 47 OPAT patients were discharged to home and facility (Nursing facility or drug rehabilitation facility), respectively.  OPAT failure (readmission within 30 days or prolonged antibiotic therapy or noncompliance or death) was 61.2% in total, 70.0% in home-OPAT group, 56.5% in facility-OPAT group. | 21 |
| Burnett^5^/  2020/  Missouri, US | Academic center (1 hospital system but unclear about the number of hospitals) | Retrospective cohort study/  266 OPAT patients | N/A | 2.5 months | Adult | N/A | N/A | 178 and 88 OPAT patients were discharged to home and facility (post-acute care facility), respectively. Readmission during OPAT happened in 18.5% of home-OPAT group and 18.2% of facility-OPAT group. | 11 |
| Certain^6^/  2022/  Utah, US | Single academic center | Quasi-experimental study/  1669 OPAT patients, all were seen by ID team and planned to have follow-up in 60 days | N/A | 3 years | Mean age 56.3 | N/A | β-lactams 71.7%  Vancomycin 31.6% | 1104 and 565 OPAT patients were discharged to home and facility (SNF), respectively.  OPAT-related 30-day readmission was 10.4% in total, 10.5% in home-OPAT group, 10.1% in facility-OPAT group. OR for readmission for facility-OPAT compared with home-OPAT was 0.96 (95%CI: 0.68-1.34). | 22 |
| Ching^7^/  2022/  US | Single academic center | Retrospective cohort study/  263 OPAT patients with substance use disorder | N/A | Unknown | Mean age 45.9 (SD 12)  Substance use disorder 100%  IVDUs 46.3% | BJI 47%  IE 20%  Other bacteremia 9% | N/A | 44 and 205 OPAT patients were discharged to home and facility (SNF), respectively. Treatment failure was 45.5% in home-OPAT group and 38.5% in facility-OPAT group. | 14 |
| D’Couto^8^/  2018/  Massachusetts, US | Single academic center | Retrospective cohort study/  52 OPAT patients who inject drugs | N/A | 6 years | Adult, Median age 30 (rage 23-51) for home group and 33 (range 24-61) for facility group  IVDUs 100% | BJI 65.4%  IE 26.9%  PJI 3.8% | Penicillins 36.5%  Cephalosporins 46.2%  Carbapenems 1.9%  Vancomycin 19.2%  Daptomycin 11.5%  FQs 1.9% | 21 and 31 OPAT patients were discharged to home and facility (SNF or rehabilitation facility), respectively.  Readmission rate was 23.1% in total, 14.3% in home-OPAT group, 29.0% in facility-OPAT group. | 18 |
| Douiyeb^9^/  2022/  Amsterdam, The Netherlands | Single academic center | Retrospective cohort study/  247 OPAT patients | N/A | 3 years | Adults  Median age 62 (IQR 50-72) | BJI 17%  IE 4%  Other bacteremia 2%  SSTI 7%  PJI 14%  Complicated UTI 14%  Respiratory infections 13%  Vascular infections 9%  IAI 7%  CNS infections 7%  Candidemia 6% | Penicillins 37%  Cephalosporins 26%  Carbapenems 11%  Vancomycin or aminoglycoside 15%  Antifungals 10% | 219 and 28 OPAT patients were discharged to home and facility (nursing facility or rehabilitation center), respectively.  30-day readmission was 10.9% in total, 9.6% in home-OPAT group, 11.3% in facility-OPAT group. | 20 |
| Felder^10^/  2016/  Oregon, US | Single academic center | Retrospective cohort study/  337 OPAT patients | N/A | 1 year 10 months | Adults  *50% were 55 years old and older.  Substance abuse 13%  Cardiovascular disease 60%  Chronic immunosuppression 13%  DM 21%  Liver disease 14%  Malignancy 14%  Mental disorder 52%  Renal disease 26%  *These numbers were for patients including 14 patients whom OPAT was asministered at an infusion center | BJI 86%  CNS infections 14% | Vancomycin 44%  β-lactams 49% | 204 and 119 OPAT patients were discharged to home and facility (SNF), respectively.  Readmission rate was 13.2% in home-OPAT group and 12.6% in facility-OPAT group. | 22 |
| Frisby^11^/  2023/  Missouri, US | Single academic center | Retrospective cohort study/  246 OPAT patients | N/A | 8 years | Adults  Median age 55 (IQR 40-65)  Cardiovascular disease 18.3%  Cirrhosis 5.3%  CKD/ESRD 10.2%  DM 28.5%  Malignancy 10.2%  Immunosuppression 14.2%  Obesity 16.3% | BJI 51.2%  IE 8.5%  SSTI 2.8%  IAI 8.9%  CNS infections 11.0%  Vascular infections 6.1%  ENT infections 2.8%  GU 4.5% PNA 3.7% | Penicillins 15.1%  Cephalosporins 47.2%  Carbapenems 16.3%  Vancomycin 33.3%  Daptomycin 3.7%  Aztreonam 0.4% Aminoglycoside 1.2%  Fluoroquinolones 1.2% | 232 and 14 OPAT patients were discharged to home and facility (SNF), respectively.  OPAT complications were 47.2% in total, 47.0% in home-OPAT group, 50.0% in facility-OPAT group. | 20 |
| Hale^12^/  2017/  New York, US | Single academic center | Retrospective cohort study/  144 OPAT patients | N/A | 6 months | Adults  Mean age 55.6 (SD 14.8) | BJI 38.9%  Other bacteremia 13.9%  SSTI 18.8%  CNS 9.7%  IAI 6.3% | Vancomycin 40.3% | 117 and 27 OPAT patients were discharged to home and facility (facility providing rehabilitation  and/or nursing services), respectively.  Drug-related problems occurred in 64.1% in home-OPAT group and 59.3% in facility-OPAT group. | 19 |
| Huang^13^/  2018/  New York, US | Single academic center | Retrospective cohort study/  200 OPAT patients | Multivariable logistic regression | 8 months | Adults  Median age 60 (range 20-95)  DM 41%  Immunosuppression 18%  Connective tissue disease 18%  Chronic pulmonary disease 17%  Peripheral vascular disease 16%  Cerebrovascular accident/dementia 10%  Liver disease 9%  Moderate/severe renal disease 4% | BJI 36%  IE 3%  Other bacteremia 5%  SSTI 24%  GU infections 18%  PNA 10%  IAI 8%  PJI 3%  CNS infection 2% | Penicillins 16%  Cephalosporins 40.5% Carbapenems 23%  Vancomycin 31%  Daptomycin 3%  Aminoglycosides 1%  Fluoroquinolones 1%  Clindamycin 1%  Metronidazole 1%  Antivirals 2% | 120 and 80 OPAT patients were discharged to home and facility (SNF or subacute rehabilitation center), respectively.  Unplanned 30-day readmission was 21% in total, 13.3% in home-OPAT group, 32.5% in facility-OPAT group.  Adjusted OR of facility-OPAT compared with home-OPAT was 3.74 (95%CI: 1.57-8.93). | 20 |
| Huck^14^/  2014/  Ohio, US | Single academic center | Retrospective cohort study/  400 OPAT patients | Multivariate logistic regression | 2 months | *Mean age 59  Charlson index score ≥3 51%  *These numbers were for patients including 18 patients whom OPAT was administered at dialysis center or infusion center | BJI 21.3%  SSTI 14.3%  IAI 15.3%  Cardiovascular infections 16.5%  CNS infections 10.5% Chest/respiratory infections 8.3%  GU infections 8.3%  Head and neck infections 1.8%  Primary disseminated infections 12.8% | β-lactams 27%  Cephalosporins 11%  Carbapenems 12%  Vancomycin 33%  Daptomycin 5%  Antifungals 6% | 238 and 144 OPAT patients were discharged to home and facility (SNF or LTAC), respectively.  Adjusted OR for readmission during OPAT for facility-OPAT compared with home-OPAT was 1.19 (95%CI: 0.64-2.21). | 20 |
| Kaul^15^/2022/New York, US | Four academic centers | Retrospective cohort study/  1759 OPAT patients | Multivariable logistic regression | 4 years | Adults  Median 66 (IQR 55-76) | BJI 26.2%  IE 5.4%  Abscess 16.3% | Penicillins 14.7%  Cephalosporins 26.7%  Carbapenems 26.8%  Vancomycin 9.5%  Daptomycin 3.0%  Antifungals 1.4%  Antiviral 1.1% | 1026 and 733 OPAT patients were discharged to home and facility (563 to subacute rehabilitation, 125 to acute rehabilitation and 65 to LTCF), respectively.  30-Day readmission was significantly lower among patients discharged to ARC with OR 0.417 (0.21-0.84) compared to patients discharged home. No significant difference for patients discharged to acute rehabilitation or LTCF. | 21 |
| Means^16^/  2016/  Illinois, US | Single academic center | Retrospective cohort study/  126 OPAT patients | N/A | 1 year 7 months | Adults  Median 55.5 (IQR 44-64)  *DM 32.9%  CKD 9.7%  Liver disease 8.8%  Malignancy 17.1%  AIDS 2.8%  Median CCI 2 (IQR 1-4)  *These numbers were for patients including 4 patients whom OPAT was administered at an infusion center | BJI 31.9%  IE 14%  SSTI 9.7%  GU 15.7%  IAI 8.3%  PNA 9.7% | β-lactams 40.2%  Vancomycin 20.8%  Daptomycin 4.6%  Fluoroquinolones 0.4% | 124 and 88 OPAT patients were discharged to home and facility (SNF or subacute rehabilitation center), respectively.  Readmission during OPAT was 21.8% in home-OPAT group, and 17.0% in facility-OPAT group. | 21 |
| Palms^17^/  2020/  Georgia, US | Two academic centers | Retrospective cohort study/  755 OPAT patients | Multivariable logistic regression | 1 year 7 months | Adults  Median age 58 (IQR 45-67)  Median CCI 2 (IQR 0-3) | BJI 44.7%  SSTI 3.4%  BSI 23.2%  CNS 6.4%  Cardiovascular 12.0%  ENT 1.5%  GI 7.8%  GU 5.4%  Lung 3.6% | Penicillins 22.1%  Cephalosporins 32.1%  Carbapenems 16.7%  Vancomycin 31.3%  Daptomycin 8.7%  Antifungals 4.4%  Fluoroquinolones 6.0%  Aminoglycosides 1.5% | 636 and 119 OPAT patients were discharged to home and facility (rehabilitation facility), respectively.  Adjusted OR for 30-day readmission for facility-OPAT compared with home-OPAT was 0.39 (95%CI: 0.20-0.73). | 21 |
| Price^18^/  2020/  Massachusetts, US | Single academic center | Retrospective cohort study/  68 OPAT patients who inject drugs | N/A | 1 year | IVDUs 100% | BJI, bacteremia/IE, Abscess | N/A | 20 and 27 OPAT patients were discharged to home and facility (SNF), respectively.  Of the 20 patients who were discharged to home, 100.0% completed the course of antibiotics, 30.0% experienced a  30-day readmission, and 15.0% relapsed. | 21 |
| Rolland^19^/  2023/  Rennes, France | Single academic center | Quasi-experimental study/  119 OPAT patients | N/A | 1 year 1 month | Adults  *Median age 66.5 (IQR 51-73)  *These numbers were for patients including 6 OPAT patients who were discharged to another hospital | *BJI 64%  IE 14%  SSTI 3%  IAI or UTI 6%  CNS 3%  Vascular infection 3% | N/A | 98 and 14 OPAT patients were discharged to home and facility (long-term care facility or rehabilitation center), respectively.  Overall, 11 patients required unplanned hospital admissions during follow-up, and three experienced  treatment failure. | 19 |
| Saini^20^/  2019/  Philadelphia, US | Single academic center | Case-control study/  266 OPAT patients | N/A | 3 years | Mean age 59.6  DM 32.2%  CKD 10.1%  Immunosuppression 12.1% | BJI 48%  IE 5.5%  Other bacteremia 9%  Abscess 8%  SSTI 7%  Device infection 5% Meningitis 3.5%  PNA, 1.3% | Penicillins 19%  Cephalosporins 38%  Carbapenems 5.5%  Vancomycin 32.5%  Daptomycin 10%  Antifungals 5%  AG 4.5%  FQ 13.5% | 194 OPAT patients who were discharged to home and 194 OPAT patients who were discharged to facility (SNF) were identified. ID outpatient follow-up within 2  weeks was associated with lower risk of all-cause 30-day readmission  (Adjusted OR, 0.33). | 19 |
| Schmidt^21^/  2017/  North Carolina, US | One healthcare system (unclear about the number or hospitals) | Retrospective cohort study/  2,228 OPAT patients | N/A | 1 year 2.5 months | Adults | Osteomyelitis 18.3%  IE 4.5%  Bacteremia 20.9%  SSTI 20.5%  Postoperative infection 19.3%  UTI 6.2% | Penicillins 7.0%  Cephalosporins 34.8%  Carbapenems 13.4%  Vancomycin 18.5%  Daptomycin 7.3% Aminoglycosides 0.9% | 1,376 and 615 OPAT patients were discharged to home and facility (SNF or rehabilitation facility), respectively.  90-day unplanned readmission rate was 18.6% in total, 15.7% in home-OPAT group, 25.0% in facility-OPAT group. | 21 |
| Townsend^22^/  2018/  Maryland, US | Two academic centers | Retrospective cohort study/  107 OPAT patients | N/A | 2 years | Adults  Mean age 54.1  IVDU 6.5%  Severe neurologic compromise 34.6%  Immunocompromise 29.0% | Musculoskeletal 54.2%  Endovascular 37.4%  CNS 8.4%  Bacteremia present 64.5%  Prosthetic material infected 47.7% | Penicillins 39.3%  Cephalosporins 14.0%  Vancomycin 45.8%  Daptomycin 2.8% | 66 and 41 OPAT patients were discharged to home and facility (SNF), respectively.  Adjusted OR for 90-day unfavorable outcomes for facility-OPAT compared with home-OPAT was 0.66 (95%CI: 0.22-1.98). | 22 |

Abbreviations: AG, aminoglycoside; AIDS, acquired immunodeficiency syndrome; BJI, bone and joint infection; BSI, bloodstream infection; CKD, chronic kidney disease; CNS, central nervous system infection; DFI, diabetic foot infection; DM, diabetes mellitus; ESRD, end stage renal disease; FQ, fluoroquinolone; GI, gastrointestinal infection; GU, genitourinary infection; HF, heart failure; IAI, intraabdominal infection; IE, infective endocarditis; OM, osteomyelitis; PJI, prosthetic joint infection; OPAT, outpatient parenteral antimicrobial therapy; OR, odds ratio; PNA, pneumonia, SA, septic arthritis; SNF, skilled nursing facility, SSTI, skin and soft tissue infection; UTI, urinary tract infection.

**1.** Allison GM, Muldoon EG, Kent DM, et al. Prediction model for 30-day hospital readmissions among patients discharged receiving outpatient parenteral antibiotic therapy. *Clinical infectious diseases : an official publication of the Infectious Diseases Society of America* 2014;58:812-819.

**2.** Barnes A, Nunez M. Diabetic Foot Infection and Select Comorbidities Drive Readmissions in Outpatient Parenteral Antimicrobial Therapy. *Am J Med Sci* 2021;361:233-237.

**3.** Brenneman E, Funaro J, Dicks K, et al. Utility of a risk assessment model in predicting 30 day unplanned hospital readmission in adult patients receiving outpatient parenteral antimicrobial therapy. *JAC Antimicrob Resist* 2023;5:dlad019.

**4.** Buehrle DJ, Shields RK, Shah N, Shoff C, Sheridan K. Risk Factors Associated With Outpatient Parenteral Antibiotic Therapy Program Failure Among Intravenous Drug Users. *Open forum infectious diseases* 2017;4:ofx102.

**5.** Burnett Y, Hamad Y. 537. Outpatient Parenteral Antimicrobial Therapy Readmissions from Post-Acute Care Facilities due to Coronavirus Disease 2019. *Open forum infectious diseases* 2020;7:S336-S336.

**6.** Certain LK, Benefield RJ, Newman M, Zhang M, Thomas FO. A Quality Initiative to Improve Postdischarge Care for Patients on Outpatient Parenteral Antimicrobial Therapy. *Open forum infectious diseases* 2022;9:ofac199.

**7.** Ching PR, George N, Kottilil S, Narayanan S. 1405. High risk or unstable substance use may predict OPAT nonadherence among people who use drugs (PWUD) admitted with invasive infections. *Open forum infectious diseases* 2022;9.

**8.** D'Couto HT, Robbins GK, Ard KL, Wakeman SE, Alves J, Nelson SB. Outcomes According to Discharge Location for Persons Who Inject Drugs Receiving Outpatient Parenteral Antimicrobial Therapy. *Open forum infectious diseases* 2018;5:ofy056.

**9.** Douiyeb S, de la Court JR, Tuinte B, et al. Risk factors for readmission among patients receiving outpatient parenteral antimicrobial therapy: a retrospective cohort study. *Int J Clin Pharm* 2022;44:557-563.

**10.** Felder KK, Marshall LM, Vaz LE, Barnes PD. Risk Factors for Complications during Outpatient Parenteral Antimicrobial Therapy for Adult Orthopedic and Neurosurgical Infections. *South Med J* 2016;109:53-60.

**11.** Frisby J, Ali N, Niemotka S, Abate G. Usefulness of Routine Laboratory Tests for Follow up of Patients Receiving Outpatient Parenteral Antimicrobial Therapy Run by Infectious Diseases Fellows. *Antibiotics (Basel)* 2023;12.

**12.** Hale CM, Steele JM, Seabury RW, Miller CD. Characterization of Drug-Related Problems Occurring in Patients Receiving Outpatient Antimicrobial Therapy. *J Pharm Pract* 2017;30:600-605.

**13.** Huang V, Ruhe JJ, Lerner P, Fedorenko M. Risk factors for readmission in patients discharged with outpatient parenteral antimicrobial therapy: a retrospective cohort study. *BMC Pharmacol Toxicol* 2018;19:50.

**14.** Huck D, Ginsberg JP, Gordon SM, Nowacki AS, Rehm SJ, Shrestha NK. Association of laboratory test result availability and rehospitalizations in an outpatient parenteral antimicrobial therapy programme. *The Journal of antimicrobial chemotherapy* 2014;69:228-233.

**15.** Kaul CM, Haller M, Yang J, et al. Assessment of risk factors associated with outpatient parenteral antimicrobial therapy (OPAT) complications: A retrospective cohort study. *Antimicrob Steward Healthc Epidemiol* 2022;2:e183.

**16.** Means L, Bleasdale S, Sikka M, Gross AE. Predictors of Hospital Readmission in Patients Receiving Outpatient Parenteral Antimicrobial Therapy. *Pharmacotherapy* 2016;36:934-939.

**17.** Palms DL, Jacob JT. Close Patient Follow-up Among Patients Receiving Outpatient Parenteral Antimicrobial Therapy. *Clinical infectious diseases : an official publication of the Infectious Diseases Society of America* 2020;70:67-74.

**18.** Price CN, Solomon DA, Johnson JA, Montgomery MW, Martin B, Suzuki J. Feasibility and Safety of Outpatient Parenteral Antimicrobial Therapy in Conjunction With Addiction Treatment for People Who Inject Drugs. *J Infect Dis* 2020;222:S494-s498.

**19.** Rolland L, Mainguy A, Boissier S, et al. A pilot project of expert nurses for the follow-up of complex intravenous antimicrobial treatment. *Infect Dis Now* 2023;53:104670.

**20.** Saini E, Ali M, Du P, Crook T, Zurlo J. Early Infectious Disease Outpatient Follow-up of Outpatient Parenteral Antimicrobial Therapy Patients Reduces 30-Day Readmission. *Clinical infectious diseases : an official publication of the Infectious Diseases Society of America* 2019;69:865-868.

**21.** Schmidt M, Hearn B, Gabriel M, Spencer MD, McCurdy L. Predictors of Unplanned Hospitalization in Patients Receiving Outpatient Parenteral Antimicrobial Therapy Across a Large Integrated Healthcare Network. *Open forum infectious diseases* 2017;4:ofx086.

**22.** Townsend J, Keller S, Tibuakuu M, et al. Outpatient Parenteral Therapy for Complicated Staphylococcus aureus Infections: A Snapshot of Processes and Outcomes in the Real World. *Open forum infectious diseases* 2018;5:ofy274.

**Supplemental Figure.** Funnel plot for readmission


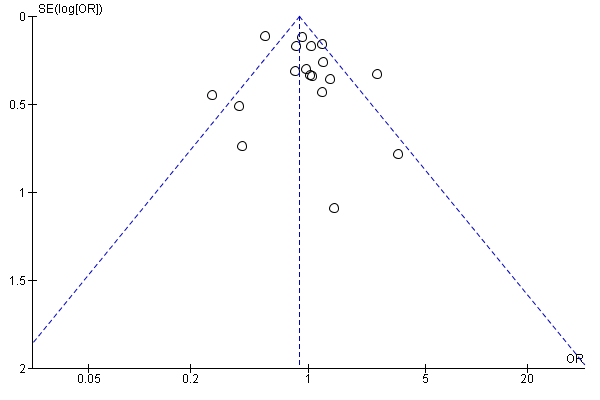

Supplement: Hasegawa et al. supplementary material [file S2732494X23004588sup001.docx]
